# The L-type voltage-gated Ca ^2+^ channel CaV1.2 regulates fibroblast-like synoviocytes invasiveness during rheumatoid arthritis

**DOI:** 10.64898/2026.10.12.758622

**Published:** 2026-10-14

**Authors:** Mark R. Tanner, R. Nicholas Elston, Madilyn J. Brandt, Brinkley A. Morse, Liang Sun, Shengwei Zou, Joseph M. Hyser, Frank T. Horrigan, Teresina Laragione, Pércio S. Gulko, Christine Beeton

## Abstract

Fibroblast-like synoviocytes (FLS) are major drivers of synovial tissue inflammation and damage during rheumatoid arthritis (RA). A hallmark of the aggressiveness of RA-FLS is their invasiveness but the molecular mechanisms that regulate this invasiveness remain poorly understood. The KCa1.1 (BK, Slo1, MaxiK, *KCNMA1* ) potassium channel is a major regulator of RA-FLS pathogenicity and inhibiting KCa1.1 expression or its activity with selective blockers reduces RA-FLS invasiveness and reduces disease severity and joint damage in rat models of RA. KCa1.1 blockade induces a Ca ^2+^ influx in RA-FLS whose intracellular signaling results in increased cell adhesion to the extracellular matrix and thus a reduction in cell migration. However, the identity of the Ca ^2+^ -permeable channel responsible for the Ca ^2+^ influx has remained elusive. Here, we demonstrate the co-expression of KCa1.1 with the L-type voltage-gated CaV1.2 channels at the plasma membrane of primary human and rat FLS. Selective blocking of the CaV1.2 channel blunts the Ca ^2+^ influx induced by KCa1.1 blockade and abrogates the effects of KCa1.1 blockade on RA-FLS invasiveness, migration, cell shape, and podoplanin expression. Whereas KCa1.1 blockers reduce disease severity in rat models of RA, treating rats with the collagen-induced arthritis model of RA with both blockers of KCa1.1 and CaV1.2 eliminates the therapeutic benefits of treatment with KCa1.1 blockers alone. Together, these data demonstrate a physical and functional coupling of KCa1.1 and CaV1.2 channels in FLS during RA and its rat model that regulate the invasive phenotype of the FLS through modulation of intracellular Ca ^2+^ homeostasis.

**Significance Statement:**

This study identifies the expression of CaV1.2 Ca ^2+^ channels in primary fibroblast-like synoviocytes isolated from the joints of patients with rheumatoid arthritis. CaV1.2 channels are functionally coupled with KCa1.1 K ^+^ channels to regulate the highly invasive phenotype of these synoviocytes, as blocking KCa1.1 induces a Ca ^2+^ influx through opening of CaV1.2 that increases the adhesion of fibroblast-like synoviocytes and inhibits their migration. Conversely, blocking CaV1.2 negates the beneficial effects of KCa1.1 on the synoviocytes *in vitro* and abrogates the therapeutics effects of a KCa1.1 blocker in reducing clinical signs of collagen-induced arthritis.

